# Whole-Body Counter(WBC) and food radiocesium contamination surveys in Namie, Fukushima Prefecture

**DOI:** 10.1371/journal.pone.0174549

**Published:** 2017-03-23

**Authors:** Yoichiro Hosokawa, Kazuki Nomura, Eiki Tsushima, Kohsei Kudo, Yuka Noto, Yoshiko Nishizawa

**Affiliations:** 1 Department of Radiation Science, Graduate School of Health Sciences, Hirosaki University, Hirosaki, Aomori, Japan; 2 Department of Comprehensive Rehabilitation Science, Graduate School of Health Sciences, Hirosaki University, Hirosaki, Aomori, Japan; 3 Department of Nursing Science, Graduate School of Health Sciences, Hirosaki University, Hirosaki, Aomori, Japan; Northwestern University Feinberg School of Medicine, UNITED STATES

## Abstract

**Purpose:**

This study examined the internal Cs exposure of residents and the Cs present in food products produced in Namie. Whole-body counter (WBC) was used for the measurement of internal exposure per each whole body of examinees.

**Methods:**

The food products which appeared to be used for consumption, were brought by residents and commercially available food items were excluded. Most of them were wild plants or food items produced by residents. Four years of data from April 2012 to March 2013 (fiscal 2012) and April 2015 to March 2016 (Fiscal 2015) were analyzed and studied.

**Results:**

The average radioactivity measured by WBC was approximately 5 Bq for Cs-134, and 20 Bq for Cs-137 and the average committed effective dose was approximately 1 *μ*Sv. The average for the residents with detectable radioactivity was 25 *μ*Sv, and the human health effects are considered to be extremely low risk. However, the radioactivity of the affected individuals showed a higher value than the theoretical attenuation rate. The majority (83.2%) of individuals exhibiting radioactivity were over 50 years old. The number of food products brought in for detection decreased as the study period progressed, but the number of food products with radioactivity had increased. While the items with a higher detection rate of radioactivity included fruits such as citron and persimmon, shiitake mushrooms exhibited the highest radioactivity. Moreover, the radioactivity of seven items in these 10 items decreased from fiscal 2012 to fiscal 2015. Mushrooms had high radioactivity and were produced over a wide area.

**Conclusion:**

We suggest that the elderly try to enjoy life and eat wild plants in moderation while inspecting food products. Therefore, we will continue to work in raising awareness of radiation and its potential presence in food products and thus the continuing necessity of monitoring radioactivity in food in the future.

## Introduction

**Fig 1:**
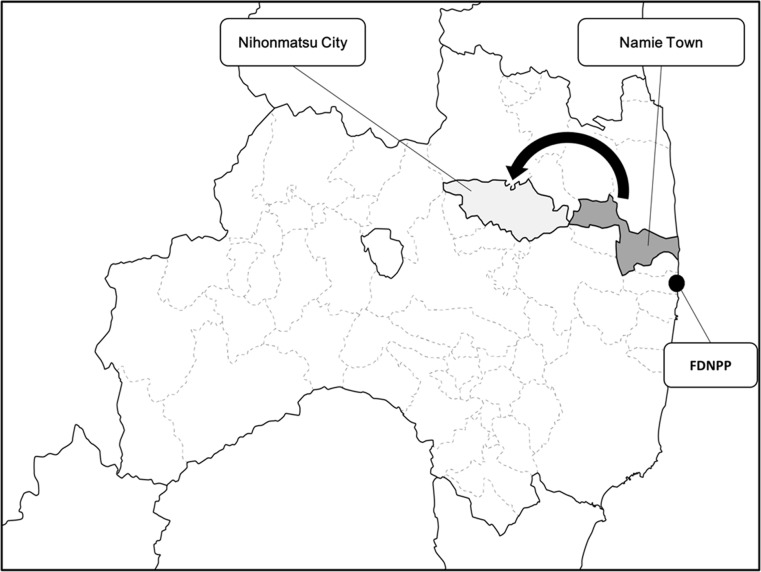
Map of Fukushima Prefecture. Residents of Namie were evacuated to other areas after the Fukushima Daiichi Nuclear Power Plant (FDNPP) accident. Residents of Nihonmatsu City were analyzed in this study.

Large amounts of artificial radionuclides such as radioiodine and radiocesium were released by the Fukushima Daiichi Nuclear Power Plant (FDNPP) accident in March 2011 [1]. Namie is located in the northern part of Fukushima Prefecture in Japan, and its population was around 21,000 when the Great East Japan earthquake struck. Namie is located about 4 km away from the power plant and the residents were evacuated considering the impact of the accident [2], (Fig 1). The exposure of the thyroid to I-131 was considered problematic at the initial stages of the accident based on the lessons learned from the Chernobyl nuclear power plant [3,4]. There have been extensive discussions on the exposure dose of the thyroid and the possibility of increased thyroid cancer rates due to the Fukushima nuclear power plant disaster. Therefore, even now thyroid screening tests are being conducted on the children of Fukushima [5,6].

A few months after the accident, the level of I-131 decreased due to its short life, and Cs-134 and Cs-137 became the main radionuclides detected in the environment around FDNPP [7]. Fortunately, Cs-134 and Cs-137 are strong γ-emitters, which allows for their fast, straightforward, and reliable detection and quantification using γ-spectroscopy. Sr-90 was also released by FDNPP, however, the maximum concentration was assumed to be 0.3% of Cs-137 concentration after April 2012 according to the report of Japan government [8]. According to the safety standards of radioactivity in foods, the acceptable level of radiocesium has been determined as 100Bq/kg by the Japanese government. Thus, the radioactivity of Cs-134 and Cs-137 is mainly estimated by a whole body counter (WBC) and a food radiation detector in order to monitor internal radiation exposure in the human body and food in Fukushima.

Several studies have assessed internal radiation exposure to radiocesium using WBC [7]. However, there are few that have discussed the internal exposure of Cs and the contamination of everyday food products [9]. This study examined the internal Cs exposure of Namie residents and the Cs present in food products produced in Namie. The number of food products brought for detection decreased as the study period progressed, but the number of food products with radioactivity had increased. While the items with a higher detection rate of radioactivity included fruits such as citron and persimmon, shiitake mushrooms exhibited the highest radioactivity. Moreover, the radioactivity of seven items in these 10 items decreased from fiscal 2012 to fiscal 2015.

## Materials and methods

Whole-body counter (WBC) tests were conducted on the residents of Namie town at the makeshift Tsushima clinic in the town of Nihonmatsu. All examinees wanted to be tested for internal exposure. Measurements were performed for 2 minutes after the initial identification of Cs using FASTSCAN (FASTSCAN™, Canberra Inc., USA). Minimum detectable amount (MDA) values of Cs-134 and Cs-137 were defined 300 Bq and 300 Bq in a 2 min measurement for the stand-up WBC, and 340 Bq and 370 Bq in a 3 min measurement for chair type WBC [10]. WBC was calibrated with the adult male-sized bottle mannequin absorption (BOMAB) phantom (American National Standards Institute, 1999) as a standard source in the USA and Canada once every three years. It was calibrated with the Canberra RMC-II (MODEL 2257) Transfer phantom (Canberra Inc., USA) and the Standard Radionuclide source (Eckert & Ziegler, Berliner, Germany), which includes Cd-109, Co-57,Ce-139, Hg-203, Sn-113, Cs-137, Y-88, Co-60 once a year. Traceability of these methods is assured by the National Institute of Standards and Technology. The difference in WBC counting efficiencies between the calibration used by the BOMAB phantom and the transfer phantom is under 10% as confimed by Momose et al. [10].

The WBC measurement value of this study is the radioactivity per each whole body. Measurements on children under 3 years old were conducted with the children standing on a 90 cm high chair. For children over 3 years old and less than 130 cm tall, measurements conducted with them standing on a 30 cm high pedestal. Residents registering radioactivity that exceeded the detection limits were asked to participate in a survey. Committed effective doses were calculated to consider to an age-appropriate dose conversion factor and residual radioactivity in human bodies [11]. Residents were informed that results of the study would be used for research before the measurements and survey.

The radioactivity of the food products was measured at the Namie town office because the residents of the town had been evacuated. The food products which were used for consumption were considered, and commercially available food items were excluded. Most of them were wild plants or food items produced by residents. Three types of instruments were used for measurement (Table 1): (CAN-OSP-NAI 8Hitachi Co., Ltd. Tokyo. Japan) was used for measurements when the resident consented to the food being crushed, and FD-08Cs1000-1-50 (Techno Co., Ltd. Osakao. Japan) was used when such consent was not obtained. When foods were measured, they were cut finely with a knife, and placed into a Marinelli plastic beaker without any gaps between food pieces. For food that could not be cut, the samples were put in an appropriate plastic box and the box was put at the center of the measuring area. Samples weighing more than 500 g were considered. SEG-EMS was used for accurate measurements when the sample weight was less than 500 g, or when the radioactivity could not be measured with simple methods.

**Table 1:** Types of machines used to survey food.

machine	measurement time	character	detector	detection limit	BG-measuring method and BG-measurement time	calibration source
CAN-OSP-NAI (Hitachi Co. Ltd. Tokyo. Japan)	30m	simplified non-destructive measurement	NaI	25Bq/kg	blank^a^:90min water^b^: 120min	Cs-137JRA^c^
FD-08Cs 1000-1-50 (Techno Co. Ltd. Osaka. Japan)	20m	simplified destructive measurement	NaI	25Bq/kg	water:40000sec	Cs-134+Cs-137JRA
SEG-EMS(Seiko Co. Ltd. Tokyo. Japan)	4000s	precision measurement	Ge semiconductor	1Bq/kg	blank: 50000sec	Co-60JRA

blank^a^: The radioactivity was measured when machine was empty.

water^b^: The radioactivity of purified water was measured by placing a plastic beaker with purified water in the machine and measuring the count.

JRA^c^: Japan Radioisotope Association.

Four years data from April 2012 to March 2013 (fiscal 2012) and April 2015 to March 2016 (Fiscal 2015) were analyzed and studied. All data were analyzed using the software SPSS 16.0J for Windows. This study was approved by the Committee of Medical Ethics of Hirosaki University Graduate School of Medicine, Hirosaki, Japan.

## Results

**Fig 2:**
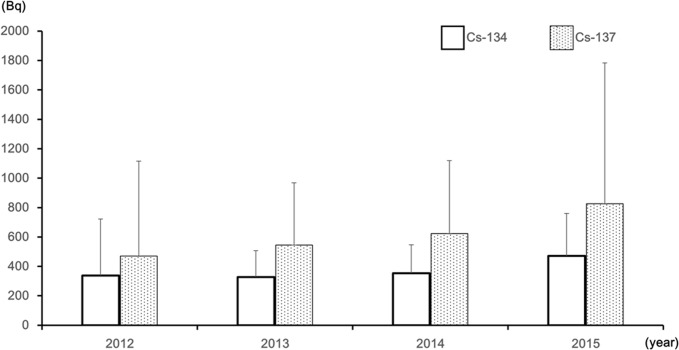
Mean value of radioactivity detected in residents with WBC measurements. Large variations were not observed for Cs-134 but a tendency of increasing radioactivity with each year was observed for Cs-137. A statistically significant difference was observed for the mean values of Cs-137 radioactivity in 2012 and 2015. [p<0.05] Significant differences were not shown between other groups.

**Fig 3:**
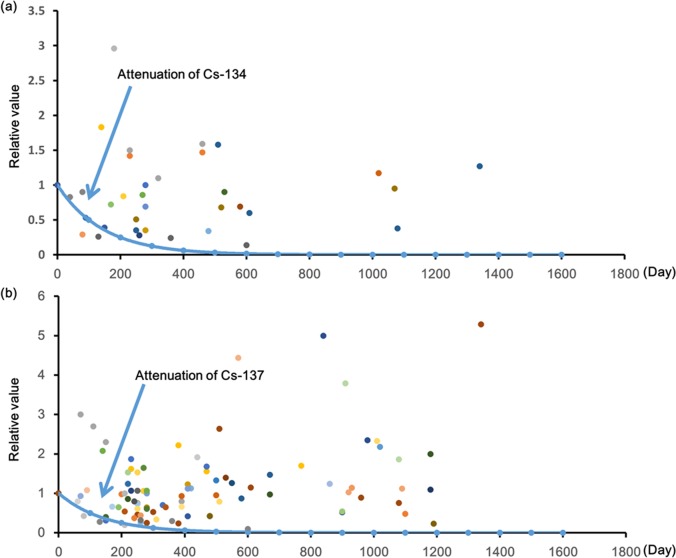
Changes in radioactivity over time for individuals detected with radioactivity. These graphs show the radioactivity attenuation ratio [Bq] for persons who underwent multiple WBC tests. For these tests, the radioactivity at the time of initial inspection is considered one. The decay curve is the curve when the mean value of the effective half-life of Cs-134, and Cs-137 is considered as 100 days. The data represents radioactivity detected in 61 and 22 persons for Cs-137 and Cs-134 respectively after multiple tests.

The number of persons tested for WBC, radioactivity, and committed effective dose from fiscal 2012 to fiscal 2015 are shown in Table 2. The overall number of persons tested, and the number of individuals detected with radioactivity both decreased with age. The average radioactivity was approximately 5 Bq for Cs-134, and 20 Bq for Cs-137 and the average committed effective dose was approximately 1 *μ*Sv. The majority (83.2%) of individuals exhibiting radioactivity were over 50 years old with men accounting for 68.2%. The average committed effective dose was 24.65 *μ*Sv for individuals detected with radioactivity. The mean value of radioactivity for individuals exhibiting radioactivity is shown in Fig 2. The trend of increasing Cs-137 radioactivity was observed for individuals detected with radioactivity, and a statistically significant difference was observed for the mean values in 2012 and 2015 with the one-way analysis of variance (p < 0.05). Cs-134 and Cs-137 attenuation rates of individuals detected with radioactivity tested two or more times was compared with the theoretical attenuation rate based on the effective half-life (100 days) to examine the cause [9]. The results are shown in Fig 3. The radioactivity of the affected individuals showed a higher value than the theoretical attenuation rate.

**Table 2:** Number of the study participants and results of whole body counter.

	total	fiscal 2012	fiscal 2013	fiscal 2014	fiscal 2015
total number (Male)	16279(7625)	7645(3590)	4570(2117)	2816(1317)	1248(601)
number of individual persons(Male)	11622(5399)	7334(3402)	4438(2053)	2774(1298)	1223(581)
average age(Male)	50.9(50.2)	50.8(49.9)	51.4(50.9)	53.3(53.2)	56.1(55.6)
committed effective dose(SD)μSv	1.0(9.3)	1.3(10.5)	0.4(4.2)	0.6(5.4)	1.0(8.9)
radioactivity mean of 134Cs(SD)Bq	5.3(60.2)	8.5(80.6)	2.2(30.6)	2.3(31.9)	3.4(46.1)
radioactivity mean of 137Cs(SD)Bq	17.1(148.2)	22.8(174.3)	7.5(80.4)	14.4(119.8)	23.1(208.8)
number of individual persons detected radioacitivity (Male)	495(342)	374(258)	61(41)	60(47)	32(21)
committed effective dose(SD)μSv	24.7(39.2)	24.6(39.0)	24.9(28.3)	25.8(25.0)	33.3(41.4)
radioactivity mean of 134Cs(SD)Bq	342.0(351.9)	337.0(385.8)	328.1(178.3)	353.9(192.3)	471.1(288.3)
radioactivity mean of 137Cs(SD)Bq	521.1(638.1)	470.4(645.2)	545.9(422.2)	622.8(496.6)	825.4(958.6)
maximum of committed effective dose μSv	502	502	125	146	192
number of 50 years old and over persons (Male)	412(281)	300(202)	54(37)	60(47)	29(19)

**Fig 4:**
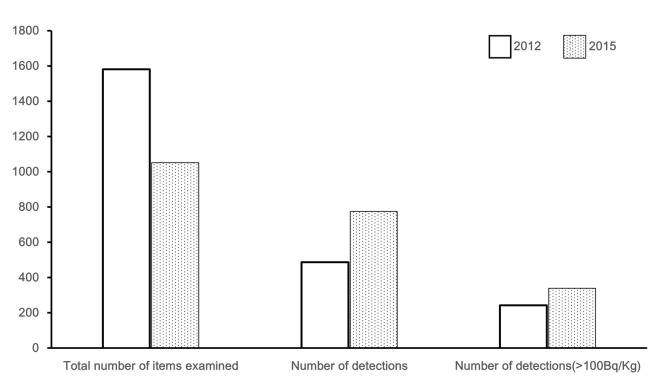
The total number of food items tested for radioactivity, detection item count, and the number of items detected with 100 Bq/kg [regulatory standard value] or more. In 2015, there was a decrease in the total number of tests, but the percentage of radioactive foods was higher than fiscal 2012.

**Fig 5:**
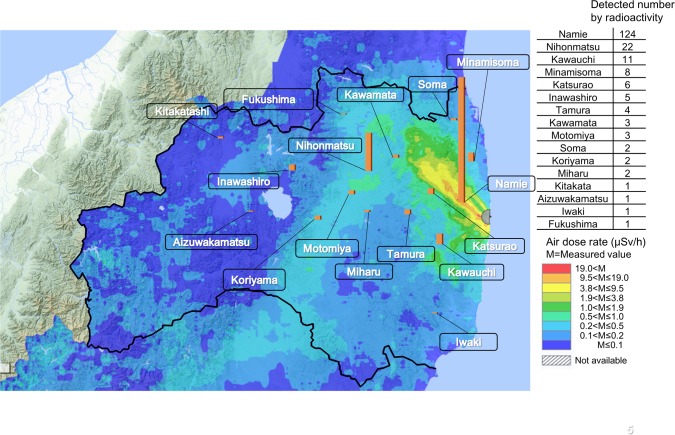
Number of radioactive mushrooms detected. Mushroom activity was higher in areas with a high external gamma dose rate.

Namie residents provided 4,542 food products for testing from 2012 to 2015 and radioactivity was detected in 2081 (45.8%). The number of food products exhibiting radioactivity was compared between fiscal 2012 and fiscal 2015 (Fig 4). The number of food products brought for detection decreased as the study period progessed, but the number of food products with radioactivity had increased. The radioactivity of Cs-134 and Cs-137 detected in the food products are shown in Table 3. Over the years, food products detected with only Cs-134 decreased, and food products exhibiting only Cs-137 increased. Also, a decreasing trend of the radioactivity ratio of Cs-134 to Cs-137 was observed, and it was assumed that Cs-134 had attenuated. The food items exhibiting high detection radioactivity rates are shown in Table 4. While the items with a higher detection rate of radioactivity included such as citron and persimmon, and shiitake mushrooms exhibited the highest radioactivity. Moreover, the radioactivity of seven items in these 10 items decreased from fiscal 2012 to fiscal 2015. Mushrooms had high radioactivity and were produced over a wide area. Fig 5 shows the number of the mushrooms detected with radioactivity on the geography. Mushroom activity was higher in areas with a high external gamma dose rate. The results of the fiscal 2013 questionnaire are shown in Table 5 because the recovery was the highest in 4 years. The persons who answered “did not care” regarding contaminated food products with radioactivity were the most in all persons who had radioactivity detected.

**Table 3:** Results of radiocesium in food.

	total	fiscal 2012	fiscal 2013	fiscal 2014	fiscal 2015
number of food items detected only Cs-134	35	29	3	3	0
number of food items detected only Cs-137	348	74	46	110	118
mean of Cs-134 radioactivity/mean of Cs-137 radioactivity	0.49	0.7	0.49	0.36	0.42

**Table 4:** Food of high detection rate.

title	subtitle	citron	persimmon	petasites	plum	bamboo shoot	bracken	shiitake mushroom	kiwi fruit	aralia Sprout	chestnut
rank		1	2	3	4	5	6	7	8	9	10
total number		199	223	189	151	137	120	104	85	82	58
detected number by radioactivity		196(98%)^a^	193(87%)	138(73%)	127(84%)	127(93%)	85(71%)	80(77%)	78(92%)	77(94%)	54(93%)
fiscal 2012	radioactivity mean±SD(Bq/kg)	1320±1255	313±596	61±70	225±323	451±758	75±49	12276±21440	333±318	309±301	1162±2822
	maximum(Bq/kg)	3160	2265	322	1098	2880	193	121000	1150	1125	10210
	minimum(Bq/kg)	12	15	11	10	15	13	19	32	29	31
	median(Bq/kg)	564	66	39	71	119	58	4070	241	148	147
fiscal 2013	radioactivity mean±SD(Bq/kg)	460±400	158±112	204±345	162±140	494±819	454±801	4999±8289	147±108	264±348	203±228
	maximum(Bq/kg)	1658	514	1099	621	2666	2550	29920	341	1270	780
	minimum(Bq/kg)	78	27	13	31	8	8	8	26	31	22
	median(Bq/kg)	335	103	38	92	186	28	973	92	67	102
fiscal 2014	radioactivity mean±SD(Bq/kg)	332±635	65±122	754±2392	93±102	319±355	467±905	4608±5646	62±72	471±679	267±432
	maximum(Bq/kg)	3943	740	14709	493	1460	3810	16460	270	2767	1130
	minimum(Bq/kg)	23	5	9	6	10	22	253	7	21	5
	median(Bq/kg)	176	36	144	68	220	118	2269	31	128	67
fiscal 2015	radioactivity mean±SD(Bq/kg)	270±445	51±50	192±297	52±42	368±538	182±224	4317±6275	53±42	711±930	157±198
	maximum(Bq/kg)	2742	274	1489	161	2364	1065	21436	146	4178	779
	minimum(Bq/kg)	16	4	5	6	7	8	11	4	14	19
	median(Bq/kg)	121	33	69	35	168	94	742	38	385	73

(%)^a^ detected number/total number.

**Table 5:** Results of questionnaire for 34 residents with detected radioactivity in fiscal 2013.

	very conscious	conscious	moderate	did not care	not care at all	total
water	5	11	0	15	3	34
rice	3	9	0	17	5	34
meat	1	11	0	17	5	34
fish	3	9	0	17	5	34
vegetable and fruit	5	10	0	17	2	34
mushroom	5	10	0	17	2	34
milk	1	11	0	16	6	34
dust^a^	3	11	1	16	3	34
during commute^b^	3	7	0	15	3	28
at work^c^	3	10	0	18	2	33

dust^a^: The number of persons who were concerned about radiation exposure from breathing dust.

during commute^b^: The number of persons who were concerned about radiation exposure during their commute.

at work^c^: The number of persons who were concerned about radiation exposure at work.

## Discussion

Residents of Namie continue to live in shelters; approximately 70% were evacuated within Fukushima and the rest outside of Fukushima. Temporary housing was set up at 30 locations in Fukushima, and as of November 2016, there were about 2,900 residents living in this temporary housing. However, the dose rate (less than 20mSv/y) has decreased with the progress in decontamination of the Namie town region, and the restrictions based on “Evacuation Directive Lift Prepared Area” and “Restricted Habitation Area” are scheduled to be lifted in March 2017. Short-term accommodations not recognized earlier were specially acknowledged as special accommodations in September 2016. We analyzed the Cs WBC measurement results of residents who were evacuated to the Nihonmatsu area as well as the radioactivity of food brought with them, which was not distributed as products.

The average radioactivity of Namie residents measured via WBC, was approximately 5 Bq for Cs-134 and 20 Bq for Cs-137. WBC results for the residents of Korosten City, Ukraine were reported as 37.2 kBq/kg [12] 10 years after the 1996 Chernobyl nuclear power plant disaster. On comparing the results obtained from the Namie residents with the residents of Korosten City, it was observed that radioactivity was much lower than that of Korosten City considering our values are systemic whole body values. Bernhardsson et al. reported on the internal and external exposure of inhabitants living in the Bryansk region of Russia between 1990–2008 [13]. They observed that, in 2008, the average effective dose [sum of external and internal exposure dose] of Cs-137 to which Chernobyl residents were exposed was estimated as 0.3 mSv/y, which corresponds to 8% and 1% of the estimated annual dose in 1990 and 1986, respectively. Hoshi reported that the mean value of annual internal dose averaged for the whole set of measurements is 0.21 mSv, and the median of the individual dose distribution is 0.12 mSv/y from 1991–1996 for children residing in the western part of Bryansk Oblast [14]. When compared to these values, the committed effective dose for Namie residents is very low; the average for all examined individuals was 1 *μ*Sv (Cs was undetectable in 95.65% of the population). The committed effective dose was also very low with an average of 24.65 *μ*Sv for individuals with detectable radioactivity.

However, both a tendency for internal exposure to increase with each passing year and a trend of radioactivity not to attenuate in the follow-up for the individuals exhibiting radioactivity was observed. Moreover, the questionnaire revealed that the individuals displaying radioactivity were mostly men over the age of 50 and were not concerned about contaminated food products. From these results, we can infer that individuals displaying radioactivity continued to eat foods contaminated with radioactivity. In a study that investigated smokers, risk perception was found to be higher in women as compared to men, and women had a higher tendency to avoid risks. [15,16]. The fact that individuals detected with radioactivity are mainly men over 50 years of age, and that they continued to eat foods with detectable radioactivity is consistent with this reasoning.

Radioactivity limits in foods were set under the Food Sanitation Act and have been in force since April 1, 2012 in Japan. Limits were set in accordance with the concept that the annual maximum permissible dose of radioactive cesium in foods should not exceed 1 mSv. Furthermore, the “limit of 100 Bq/kg” for general foods was determined by choosing the most rigorous limits among the calculated values [17]. We got the impression that the limits were understood by the residents when we spoke to them. The increase in food products with detectable radioactivity over time, despite the decrease in the number of food products brought in for inspection was due to an increasing awareness among residents of possibly radioactive plants within their collection region.

The overall radioactivity of food products decreased in our study because the half-life of Cs-134 is 2 years. However, there were also many food items that exceeded the limits. Mushrooms, in particular, were found to be radioactive. Based on the 1986 Chernobyl Nuclear Power Plant, it is well known that radiocesium tends to concentrate in wild mushrooms [18]. Nakashima found that radioactive cesium exceeding 100 Bq/kg was detected in 125 of 154 mushrooms (81.2%) in Kawauchi Village in Fukushima. They calculated committed effective doses based on 6,278 g per year of mushrooms per year, the average intake of Japanese citizens (age > 20 years, 17.2 g/day), ranging from doses of 0.11–1.60 mSv. [19]. In our study, some mushrooms from areas with a high external gamma dose rate were tested. The residents of this region were accustomed to eating wild plants with foraging being one of the favorite pastimes of the elderly. This study is a observational study, and though we cannot say for sure, we feel that the elderly eat wild plants in moderation while inspecting the food products and trying to enjoy life. Therefore, we will continue to work for raising awareness of radiation and its potential presence in food products and thus the continuing necessity of monitoring radioactivity in food in the future.

In conclusion, the committed effective dose for internal exposure to Cs for residents was, on an average, 1 *μ*Sv. The average for the residents with detectable radioactivity was 25 *μ*Sv, and the human health effects are considered to be extremely low risk. While trend of decreasing radioactivity has been observed in the food items brought in for inspection, but even now, food items such as mushrooms show a high level of radioactivity. WBC testing and food inspection should continue in the future in order to monitor radioactivity of residents in Namie.

## Supporting information

S1 Table Measurement result of WBC. (DOCX)

S2 Table Radioactivity Measurements of food. (DOCX)
