# Supplementary material for: Whole-Body Counter(WBC) and food radiocesium contamination surveys in Namie, Fukushima Prefecture
Source: PLoS One. 2017 Mar 23;12(3):e0174549. doi: 10.1371/journal.pone.0174549 (PMC5363944; doi:10.1371/journal.pone.0174549)
Supplement: S1 Table — (DOCX) [file pone.0174549.s001.docx]

S1 Table Measurement result of WBC

| test day | No. | gender | date of birth | age at test date | measurement result | | | | intake at the start of age | radioactivity | | committed effective dose |
| --- | --- | --- | --- | --- | --- | --- | --- | --- | --- | --- | --- | --- |
|  |  |  |  |  | body surface | measurement result of WBC | | |  |  |  |  |
|  |  |  |  |  |  | pedestal use | ^134^Cｓ | ^137^Cｓ |  | ^134^Cｓ | ^137^Cｓ |  |
|  |  |  |  |  | (cpm) |  | (kBq） | (kBq） |  | (Bq） | (Bq） | (mSv） |
| 2015/4/1 | 975 | female | 1968/10/31 | 46 | ND |  | 1.700E-01 | 2.300E-01 | 42 | ND | ND | 0.000 |
| 2015/4/1 | 976 | female | 2001/10/22 | 13 | ND |  | 1.700E-01 | 2.300E-01 | 9 | ND | ND | 0.000 |
| 2015/4/1 | 4105 | female | 1926/3/16 | 89 | ND |  | 1.700E-01 | 2.300E-01 | 84 | ND | ND | 0.000 |
| 2015/4/1 | 4491 | female | 1969/3/18 | 46 | ND |  | 1.700E-01 | 2.300E-01 | 41 | ND | ND | 0.000 |
| 2015/4/1 | 4526 | male | 2002/3/13 | 13 | ND |  | 1.700E-01 | 2.300E-01 | 8 | ND | ND | 0.000 |
| 2015/4/1 | 4539 | male | 2004/3/16 | 11 | ND |  | 1.700E-01 | 2.300E-01 | 6 | ND | ND | 0.000 |
| 2015/4/1 | 4541 | female | 1927/9/18 | 87 | ND |  | 1.700E-01 | 2.300E-01 | 83 | ND | ND | 0.000 |
| 2015/4/1 | 8102 | female | 1976/6/5 | 38 | ND |  | 1.700E-01 | 2.300E-01 | 34 | ND | ND | 0.000 |
| 2015/4/1 | 8110 | female | 2007/6/20 | 7 | ND | 30cm | 1.700E-01 | 2.300E-01 | 3 | ND | ND | 0.000 |
| 2015/4/1 | 8119 | male | 2009/8/26 | 5 | ND | 30cm | 1.700E-01 | 2.300E-01 | 1 | ND | ND | 0.000 |
| 2015/4/1 | 8125 | male | 1974/10/19 | 40 | ND |  | 1.700E-01 | 2.300E-01 | 36 | ND | ND | 0.000 |
| 2015/4/2 | 9359 | male | 1993/1/29 | 22 | ND |  | 1.700E-01 | 2.300E-01 | 18 | ND | ND | 0.000 |
| 2015/4/2 | 9362 | male | 1961/3/19 | 54 | ND |  | 1.700E-01 | 1.510E-01 | 49 | ND | ND | 0.000 |
| 2015/4/3 | 4400 | male | 1949/8/8 | 65 | ND |  | 1.700E-01 | 2.300E-01 | 61 | ND | ND | 0.000 |
| 2015/4/3 | 4402 | female | 1957/1/2 | 58 | ND |  | 1.700E-01 | 2.300E-01 | 54 | ND | ND | 0.000 |
| 2015/4/3 | 4408 | female | 1982/6/22 | 32 | ND |  | 1.700E-01 | 2.300E-01 | 28 | ND | ND | 0.000 |
| 2015/4/3 | 6813 | male | 1948/3/1 | 67 | ND |  | 1.700E-01 | 2.877E-01 | 63 | ND | 290 | 0.010 |
| 2015/4/7 | 1175 | male | 1958/4/23 | 56 | ND |  | 1.700E-01 | 2.300E-01 | 52 | ND | ND | 0.000 |
| 2015/4/7 | 5461 | female | 1944/3/14 | 71 | ND |  | 1.700E-01 | 2.300E-01 | 66 | ND | ND | 0.000 |
| 2015/4/7 | 5500 | male | 1944/3/30 | 71 | ND |  | 1.700E-01 | 2.300E-01 | 66 | ND | ND | 0.000 |
