# Supplementary material for: Whole-Body Counter(WBC) and food radiocesium contamination surveys in Namie, Fukushima Prefecture
Source: PLoS One. 2017 Mar 23;12(3):e0174549. doi: 10.1371/journal.pone.0174549 (PMC5363944; doi:10.1371/journal.pone.0174549)
Supplement: S2 Table — (DOCX) [file pone.0174549.s002.docx]

S2 Table adioactivity Measurements of food.

| test day | № | sampling point | item | weight(kg) | machine | Cs134 | Cs137 | 134Cs/137Cs | Total of Cs | specific radioactivity（Ｂｑ/Ｋｇ） |
| --- | --- | --- | --- | --- | --- | --- | --- | --- | --- | --- |
| 2015/4/1 | 1 | Nihonmatsu | butterbur | 0.357 | S | - | - | - | 15 | 43 |
| 2015/4/1 | 2 | Nihonmatsu | butterbur | 0.287 | C | ND | ND | - | ND | - |
| 2015/4/2 | 4 | Fukushima | green onion | 0.569 | F | - | - | - | 7 | 13 |
| 2015/4/2 | 5 | Nihonmatsu | onion | 1.092 | F | - | - | - | ND | - |
| 2015/4/2 | 3 | Otama | green onion | 0.502 | F | - | - | - | ND | - |
| 2015/4/3 | 6 | Nihonmatsu | butterbur | 0.295 | S | - | - | - | 12 | 42 |
| 2015/4/3 | 7 | Nihonmatsu | butterbur | 0.298 | C | ND | ND | - | ND | - |
| 2015/4/6 | 8 | Namie | butterbur | 0.493 | S | - | - | - | 59 | 120 |
| 2015/4/6 | 9 | Namie | butterbur | 0.414 | S | - | - | - | 217 | 523 |
| 2015/4/7 | 17 | Namie | butterbur | 0.399 | C | 72 | 229 | 0.31 | 301 | 754 |
| 2015/4/7 | 16 | Namie | butterbur | 0.528 | C | 14 | 49 | 0.28 | 63 | 119 |
| 2015/4/7 | 12 | Namie | shiitake mushroom | 0.916 | C | 96 | 375 | 0.26 | 470 | 513 |
| 2015/4/7 | 10 | Nihonmatsu | lily bulb | 0.340 | S | - | - | - | 74 | 218 |
| 2015/4/7 | 11 | Namie | shiitake mushroom | 1.037 | F | - | - | - | 589 | 568 |
| 2015/4/7 | 13 | Namie | aralia sprout | 0.083 | S | - | - | - | 342 | 4117 |
| 2015/4/7 | 15 | Namie | aralia sprout | 0.188 | S | - | - | - | 854 | 4543 |
| 2015/4/7 | 14 | Namie | aralia sprout | 0.145 | S | - | - | - | 1221 | 8423 |
| 2015/4/8 | 18 | Nihonmatsu | chinese cabbage | 0.798 | F | - | - | - | ND | - |
| 2015/4/8 | 19 | Nihonmatsu | chinese cabbage | 0.708 | F | - | - | - | ND | - |
| 2015/4/9 | 20 | Namie | fiddlehead fern | 0.062 | C | 329 | 1150 | 0.29 | 1479 | 23855 |

machine (S:SEG-EMS, C:CAN-OSP-NAI, F:FD-08Cs 1000-1-50)
